# Beyond reversal: ubiquitin and ubiquitin-like proteases and the orchestration of the DNA double strand break repair response

**DOI:** 10.1042/BST20190534

**Published:** 2019-11-26

**Authors:** Alexander J. Garvin

**Affiliations:** Institute of Cancer and Genomic Sciences, University of Birmingham, Edgbaston, Birmingham, U.K.

**Keywords:** DNA synthesis and repair, double strand break, DUB, SENP, sumoylation, ubiquitin

## Abstract

The cellular response to genotoxic DNA double strand breaks (DSBs) uses a multitude of post-translational modifications to localise, modulate and ultimately clear DNA repair factors in a timely and accurate manner. Ubiquitination is well established as vital to the DSB response, with a carefully co-ordinated pathway of histone ubiquitination events being a central component of DSB signalling. Other ubiquitin-like modifiers (Ubl) including SUMO and NEDD8 have since been identified as playing important roles in DSB repair. In the last five years ∼20 additional Ub/Ubl proteases have been implicated in the DSB response. The number of proteases identified highlights the complexity of the Ub/Ubl signal present at DSBs. Ub/Ubl proteases regulate turnover, activity and protein–protein interactions of DSB repair factors both catalytically and non-catalytically. This not only ensures efficient repair of breaks but has a role in channelling repair into the correct DSB repair sub-pathways. Ultimately Ub/Ubl proteases have essential roles in maintaining genomic stability. Given that deficiencies in many Ub/Ubl proteases promotes sensitivity to DNA damaging chemotherapies, they could be attractive targets for cancer treatment.

## Introduction

**Figure 1.**
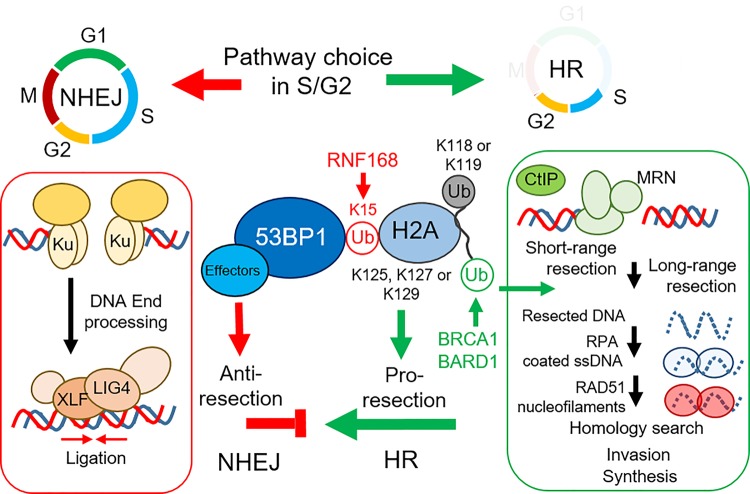
NHEJ occurs at all stages of the cell cycle. Ku70/80 heterodimers bind and stabilise the ends of the DSB. Other factors are also recruited by the Ku dimer. DNA end processing chemically modifies the bases to allow re-ligation. Ligase 4 (LIG4), XLF and other repair factors ligate the broken DNA ends. HR is restricted to S/G2, initial DNA 3′ end resection is initiated by the MRN complex and CtIP. Further resection promoted by the BRCA1–BARD1 heterodimer, EXO1 and DNA2 promote long range resection. The ssDNA generated by end resection is coated in the RPA heterotrimeric complex, which is then displaced by RAD51 nucleofilaments with the aid of the BRCA1–PALB2–BRCA2 complex. After RAD51 filament formation, homology search followed by invasion of the sister chromatid and synthesis of new DNA occurs. As the biology of these later processes in the context of Ub/Ubl modifications is poorly understood they are not illustrated here. As NHEJ can also occur in S/G2 pathway choice between HR and NHEJ occurs. The pro-NHEJ factor 53BP1 is recruited by K15 Ub modified H2A/H2AX while modification of K125/K127 or K129 by mono-Ub aids in nucleosome remodelling and displacement of 53BP1, this favours DNA end-resection and HR. Thus maintaining the balance of Ub signals dictates repair pathway choice.

DNA double stranded breaks are highly toxic lesions that occur when both strands of DNA are broken. The breaks can be formed from endogenous processes such as DNA replication, or exogenous sources such ionising radiation. Two major pathways repair double strand breaks (DSBs) (Figure 1). Non-Homologous End Joining (NHEJ) occurs throughout the cell cycle and uses small regions of homology present in DNA overhangs followed by direct re-ligation for repair. If the overhangs are not compatible loss of nucleotides can lead to mutations. The second pathway Homologous Recombination (HR) is restricted to late S/G2 phases of the cell cycle as it relies on homologous DNA sequences, provided by sister chromatids, for homology directed repair. HR repair is generally error free [1]. Ub plays essential roles in orchestrating both repair pathways. A central function of Ub in DSB repair is maintaining the balance between NHEJ and HR in S/G2 cells. RNF168 mono-ubiquitination on K15 of H2A or H2AX promotes the recruitment of the pro-NHEJ factor 53BP1, while BRCA1–BARD1 mediated mono-ubiquitination of H2A K125/K127 or K129 promotes displacement of 53BP1 and subsequent DNA end-resection, the essential precursor step for HR [2]. Maintaining the correct balance is critical, as too much NHEJ can be mutagenic, but un-restrained DNA end-resection is also toxic. To co-ordinate DSB repair Ub and the Ubl SUMO and NEDD8 play multiple roles, they promote the recruitment and retention of repair factors at damaged chromatin, alter protein–protein interactions and enable clearance and termination of repair signalling [3,4]. As all of these processes require exquisite temporal and spatial tuning, a plethora of Ub/Ubl proteases are deployed by DNA damage signalling to ensure efficient and accurate DSB repair [5]. The profound consequences that result from the mis-regulation of these proteases offers fascinating insight into the intricacies of DSB repair.

## Ku70/80 and the MRN complex

**Figure 2.**
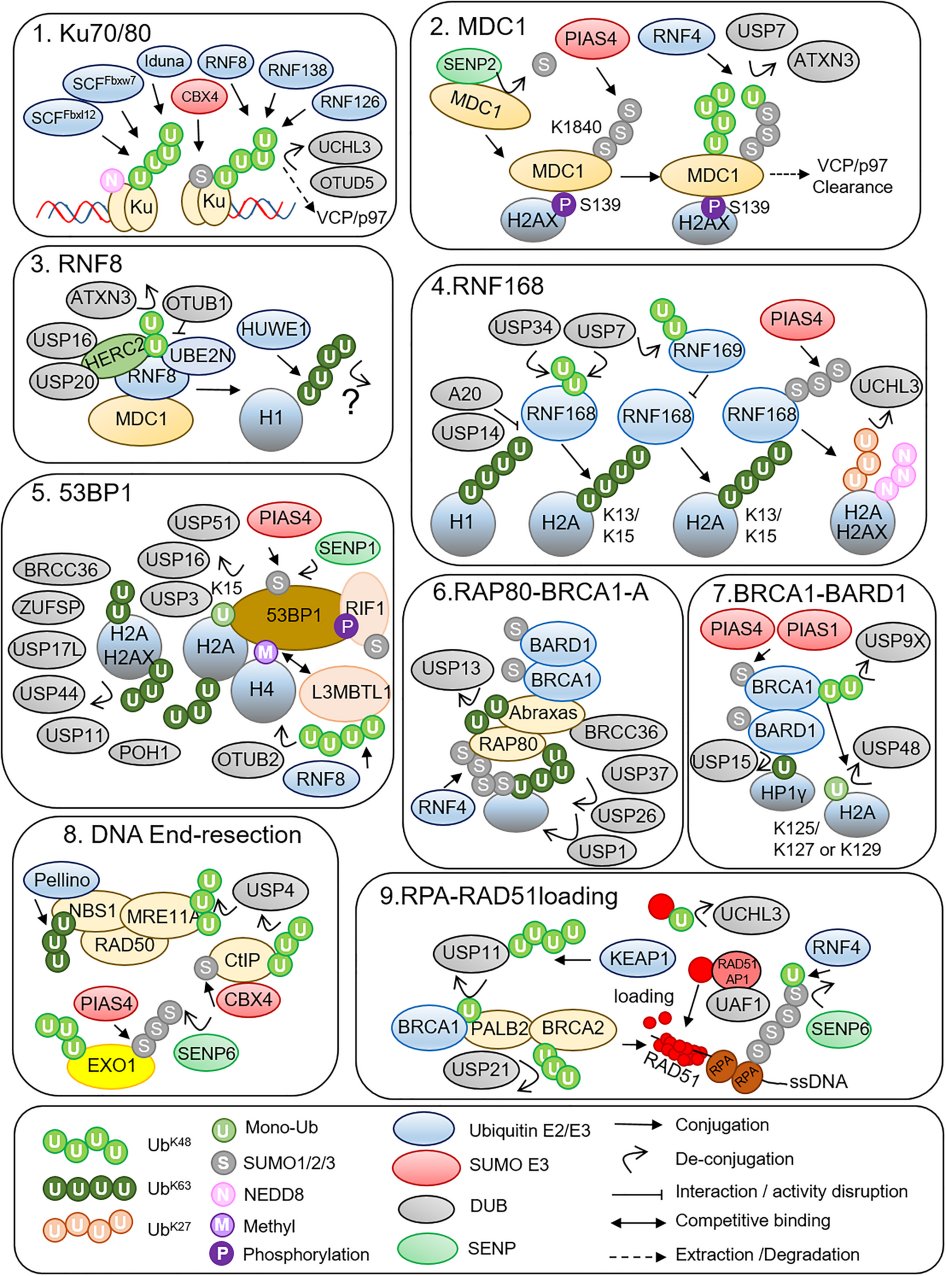
The DNA bound Ku70/80 heterodimer is ubiquinated and SUMOylated by multiple E3 ligases. Ultimately this signals for Ku70/80 dimer removal from chromatin by VCP/p97. MDC1 is constitutively protected from excessive SUMOylation through its interaction with SENP2. This interaction is disrupted by ATM signalling which allows the DSB located MDC1 to be hyperSUMOylated by PIAS4 which also localises to DSBs. The SUMOylated MDC1 is then recognised by the SUMO directed Ub ligase RNF4 which ubiquitinates MDC1 and promotes extraction from chromatin by VCP. The RNF4 step can be countered by ATXN3, slowing MDC1 turnover at DSBs. RNF8 is recruited by MDC1 where it forms K63-Ub linkages on H1.2. HUWE1 also contributes to the generation of Ub chains on H1.2. OTUB1 inhibits RNF8 activity by disrupting Ub recognition by the E2 UBE2N. Conversely, HERC2 stimulates RNF8–UBE2N activity in a SUMOylation dependent manner. HERC2 also interacts with two DUBs, USP16 and USP20 both of which are involves in DSB repair signalling. ATXN3 counters RNF8 auto-ubiquitination. RNF168 interacts with RNF8 generated H1.2^K63-Ub^ and amplifies this Ub signal through recognition of its own Ub product — K15 mono-ubiquitinated H2A or H2AX. Two DUBs, (A20 and USP14) interfere with RNF168's initial recruitment to H1.2. Two more DUBs (USP7 and USP34) are needed to protect RNF168 from auto-degradation. The paralog RNF169 competes with RNF168 for H2A/H2AX^K15-Ub^ binding. 53BP1 recognises the K15-Ub modified H2A or H2AX generated by RNF168. Additionally, 53BP1 is recruited by H4K20me2, a modification that is competitively bound by L3MBTL1. Ubiquitination and extraction from chromatin of L3MBTL1 is required for efficient 53BP1 spreading along chromatin. The turnover of L3MBTL1 is antagonised by OTUB2. Multiple DUBs regulate the spread of 53BP1 via regulating the amplitude of Ub/Ub^K63^ at DSBs, some of these DUBs directly counter the H2A/H2AX^K15-Ub^ modification while others may act on other uncharacterised K63-Ub modified proteins. RAP80 and the BRCA1-A complex are recruited to DSBs in a K63-Ub dependent fashion, although RAP80 also recognises SUMO modifications (most likely generated by RNF4), suggesting that the RAP80–BRCA1-A complex may recognise a different signal to 53BP1. BRCA1 recruitment into the BRCA1-A complex is thought to divert its activity from promoting DNA end-resection. At least two DUBs (USP26 and USP37) specifically counter the Ub signal that promotes RAP80–BRCA1 accrual. USP13 trims Ub from the RAP80 UIM domain to allow efficient Ub recognition. BRCA1–BARD1 also recruit to DSBs independently of K63-Ub signalling. Ubiquitination inhibits the BARD1- HP1γ interaction needed for efficient accumulation of BRCA1–BARD1 at DSBs. This disruptive ubiquitination event is countered by USP15. The ubiquitination and turnover of BRCA1 is countered by USP9X. BRCA1–BARD1 also generates mono-Ub on the far C terminus of H2A (K125, K127 or K129). This signal promotes DNA end-resection and aids in the displacement of 53BP1 from DSBs. USP48 restricts the spread of H2A^K125/127/129-Ub^ preventing excessive DNA end-resection. The MRN complex and CtIP, both of which are essential for the early steps in DNA end-resection are ubiquitinated, USP4 antagonises these modifications. SUMOylation promotes degradation of the DNA exonuclease EXO1, to control DNA end-resection which is countered by SENP6. The loading of the RPA hetero-trimer onto single stranded DNA (generated through DNA end-resection) and its exchange with RAD51 are essential steps in HR. The RPA70 subunit is SUMOylated and degraded by RNF4, this is countered by SENP6. Loading of RAD51 onto ssDNA is promoted by BRCA2 in the BRCA1–PALB2–BRCA2 complex. This complex can only form due to the activity of USP11 which removes an inhibitory Ub modification that is disruptive to BRCA1–PALB2 interactions. USP11 levels are cell cycle regulated by KEAP1. USP1–UAF1 non-catalytically aids in the loading of RAD51, UCHL3 de-ubiquitinates RAD51 promoting its ability to load onto DNA, while USP21 protects the unstable BRCA2 protein from degradation.

DSBs are rapidly detected by two complexes, the Ku70/Ku80 heterodimer which promotes NHEJ and MRN (MRE11A/RAD50/NBS1) which, with the exonuclease CtIP promotes HR [6,7]. Multiple Ub-E3 ligases ubiquitinate Ku heterodimers which ultimately results in extraction by the VCP/p97 complex [8,9]. The importance of this regulation is highlighted by the toxic effects of trapped Ku heterodimers which not only prevent NHEJ repair termination but also block the early steps in HR repair [9]. Ubiquitination of Ku heterodimers is countered by at least two DUBs (De-ubiquitinating proteases). UCHL3 de-ubiquitinates and stabilises Ku80 which is required for Ku80 retention at DSBs [10]. OTUD5 also stabilises Ku80, loss of OTUD5 reduces NHEJ repair efficiency [11] (Figure 2).

The MRN complex performs several activities, including DNA unwinding, promotion of DNA end resection and recruitment of the master DSB kinase ATM to the DSB [6]. ATM phosphorylates multiple DSB repair proteins, including histone H2AX at Ser^139^ (γH2AX) [12]. Pellino generates K63-Ub chains on NBS1 promoting ATM activation and HR repair, suggesting that Ub signalling also regulates MRN activity [13]. Indeed, USP4 is recruited to DSBs through interaction with NBS1 where it also regulates CtIP ubiquitination [14].

## MDC1 (Mediator of Damage Checkpoint 1)

MDC1 interacts with γH2AX via its BRCT repeats [15], and has multiple roles in DSB signalling, it aids in the retention of ATM which further propagates the γH2AX signal along damaged chromatin and it recruits RNF8 [16,17]. While MDC1 recruitment is phosphorylation dependent, turnover at DSBs in G1 requires SUMOylation and ubiquitination. Following recruitment to DSBs MDC1 is SUMOylated by PIAS4 [18–22]. MDC1 SUMOylation promotes recognition by RNF4, an Ub-E3 that interacts with SUMOylated substrates [23]. This in turn promotes VCP/p97 dependent extraction of MDC1 from DSBs [20–22,24]. The basal SUMOylation state of MDC1 is maintained by the SUMO protease SENP2 which dissociates from MDC1 shortly after DSB induction. This allows un-restrained SUMOylation of MDC1 by PIAS4 on DSBs which ultimately triggers MDC1 removal by VCP. In the absence of SENP2 the constitutively hyperSUMOylated MDC1 undergoes premature PIAS4-RNF4-VCP dependent clearance from DSBs resulting in ablated RNF8 accrual and K63-Ub signalling [24]. The RNF4 dependent clearance of MDC1 is antagonised by ATXN3, a DUB that is rapidly recruited to DSBs by SUMO [25]. USP7 also interacts with and stabilises the MDC1–MRN complex [26].

## RNF8

RNF8 is recruited to DSBs via its FHA domain that recognises phosphorylated MDC1 [27], where along with its partner E2 (UBE2N) K63-Ub chains are conjugated on histone H1 [28]. HUWE1 primes the ubiquitination of H1 prior to extension by RNF8 [29]. Several DUB dependent modes of RNF8 regulation have been identified. OTUB1 blocks the generation of K63-Ub through a non-enzymatic activity that involves disruption of the RNF8–UBE2N catalytic cycle [30,31]. The RNF8–UBE2N complex is also regulated by HERC2 which stimulates the generation of K63-Ub at DSBs by stabilising or promoting the interaction of RNF8 with UBE2N [32]. This activity is promoted by SUMOylation of HERC2 through PIAS4 [33]. HERC2 interacts with USP20, a DUB that is recruited to DSBs and is involved in checkpoint signalling in response to replication stress and HR repair [34–37]. HERC2 also interacts with and stabilises USP16 which is involved in H2A de-ubiquitination in DSB repair [38]. RNF8 auto-ubiquitination is countered by ATXN3, which is required to balance VCP/p97 dependent extraction from damaged chromatin [39].

## RNF168

H1^K63-Ub^ is recognised by a MIU domain of RNF168, which conjugates mono-ubiquitin to H2A or H2AX at K15 [28,40]. This serves as a docking site for the resection antagonist/pro-NHEJ factor 53BP1 [41,42]. A second MIU domain in RNF168 also recognises its own Ub product, allowing spreading and amplification of H2A/H2AX-Ub^K15^ [28]. RNF168 is de-stabilised following DSB induction and at least two DUBs, USP34 and USP7 are involved in countering RNF168 turnover. Loss of either DUBs results in reduced RNF168 and less K63-Ub signalling at DSBs [43,44]. RNF168 accumulation at damaged chromatin is also regulated by DUBs. A20 interacts with RNF168 upon DSB induction and disrupts recruitment to H1^K63-Ub^ non-catalytically [45]. USP14 also recruits to DSBs where it interacts with RNF168 via its MIU1 domain and impairs RNF168 Ub signalling [46].

USP3, USP51 and USP16 de-ubiquitinate H2A/H2AX^K15-Ub^ with varying degrees of specificity [47–49]. Loss of these DUBs causes excessive spreading of H2A/H2AX^K15-Ub^ and its downstream reader 53BP1. In addition to K63-Ub, RNF168 generates K27-Ub conjugates, which are required for the recruitment of 53BP1 and RAP80–BRCA1 [50]. Although the biology of K27-Ub in DSB repair is not well understood, it has since been shown that RNF168 generated K27-Ub conjugates can be antagonised by UCHL3 [51].

H2A/H2AX^K15-Ub^ is also recognised by the RNF168 paralog RNF169 which competes with 53BP1. RNF169 therefore limits the recruitment of the pro-NHEJ/anti-resection factor 53BP1 and promotes HR repair [41,52–55]. Like RNF168, RNF169 is also stabilised by USP7 [56].

Several DUBs were characterised for their roles in DSB signalling prior to the identification of H2A/H2AX^K15-Ub^ generated by RNF168 and therefore it is not clear if they only target this modification, other sites on H2A/H2AX or non-histone K63-Ub modified proteins. USP44, USP17L2/DUB3, USP11, ZUFSP and POH1 loss promotes the excessive spreading of Ub at DSBs and concomitant excessive accumulation of 53BP1 [57–65].

## H2a-K118/K119

H2A is predominantly mono-ubiquitinated at K118/K119 by BMI1:RING1B E3 ligase. H2A^K118/K119-Ub^ has important, but not fully understood the role in DSB repair [66]. BAP1 is the dominant H2A^K118/K119-Ub^ DUB [67] and is rapidly and transiently recruited to DSBs [37,68,69]. BAP1 loss specifically reduces HR but not NHEJ [68,69].

## 53BP1

53BP1 is a major adapter protein that recruits to DSBs via histone phosphorylation [70], methylation [71] and ubiquitination [42]. While 53BP1 has no enzymatic activity it does recruit a number of other proteins that antagonise DNA end-resection and subsequent HR pathways mediated by BRCA1. The major effectors of 53BP1 include PTIP, RIF1 and the Rev7-Shieldin-CST complexes [72].

The Tudor domain of 53BP1 recognises H4K20me2, which is also recognised with higher avidity by L3MBTL1 and JMJD2A [73,74]. To allow maximal 53BP1 recruitment these factors need to be displaced from chromatin. L3MBTL1 is K48 ubiquitinated by RNF8 and extracted by VCP/p97 from chromatin [73]. This ubiquitination is antagonised by OTUB2, a DUB that is recruited to DSBs and slows L3MBTL1 Ub dependent removal from chromatin. This limits excessive 53BP1 spreading [37,75]. SUMOylation at DSBs is also important for 53BP1 recruitment, and 53BP1 is itself SUMOylated by PIAS4 in G1 [18,76]. SUMOylation of 53BP1 is antagonised by SENP1, as displacement of SENP1 from nuclear pores reduces 53BP1 SUMOylation and disrupts NHEJ repair [77]. The 53BP1 interacting partner RIF1 is also PIAS4 SUMOylated with similar kinetics to 53BP1 [76].

## RAP80 and the BRCA1-A complex

RAP80 is a reader of K63-Ub polymers at DSBs and serves as a docking module for the BRCA1-A complex [78–81]. In addition to K63-Ub, RAP80 interacts with SUMO chains, likely through its recognition of mixed K63-Ub-SUMO linkages for which it has an eighty times higher binding preference over K63-Ub chains. RNF4 is the likely source of these linkages due to its localisation at DSBs and ability to generate mixed K63-Ub-SUMO conjugates [82,83].

BRCC36 (BRCA1/2 Containing Complex 36) was the first DUB characterised in DSB repair [84] and resides within the BRCA1-A complex. BRCC36 de-ubiquitinates H2A/H2AX and RAP80. Loss of BRCC36 results in extensive spreading of K63-Ub at DSBs, suggesting its role is to limit the amplitude of K63-Ub at DSBs [85,86]. BRCA1-A components, including BRCC36 are antagonists of HR repair. The BRCA1-A complex has been proposed to divert BRCA1–BARD1 Ub ligase activity to dampen DNA end-resection. Loss of BRCA1-A components causes a hyper-resection phenotype [87,88]. USP26 and USP37 are both recruited to DSBs and act on conjugates generated by RNF168. However, these DUBs act on the RAP80 pathway rather than the 53BP1 pathway. USP26/USP37 depletion increases size but not number of BRCA1 foci. This excessive spreading of BRCA1 can be countered by depletion of RAP80, suggesting the BRCA1 is being recruited into enlarged BRCA1-A complexes. In cells depleted of USP26/USP37, BRCA1 interacts less efficiently with PALB2, which bridges interaction with BRCA2. As BRCA2 is required for RAD51 loading this is a likely cause of failed HR in these cells [57,58]. The K63-Ub dependent interaction by RAP80 is disrupted by ubiquitination of lysines in close proximity to its UIM domain. USP13 counters this modification to enable recruitment of RAP80 and the BRCA1-A complex to DSBs [89]. BRCA1 recruitment in S/G2 is also regulated by USP1–UAF1, which antagonises K63-Ub chains that serve as recruiters for BRCA1. Unlike several other DUBs that antagonise K63-Ub, over-expression of USP1 only affected BRCA1 recruitment and not 53BP1. USP1 is locally inactivated by K11-Ub modification generated by APC^Cdh1/Ube2S^ which is also localised to DSBs. The APC^Cdh1^ dependent inactivation of USP1 thus removes the restraint on K63-Ub generation in a cell cycle dependent manner [90].

## BRCA1–BARD1

BARD1 aids in the recruitment of its heterodimeric partner BRCA1 to DSBs through a number of different interacting partners, including HP1γ [91]. The BARD1 BRCT repeats are modified by K63-Ub which disrupts interaction with HP1γ and therefore localisation of BRCA1–BARD1 to DSBs. This inhibitory ubiquitination is removed by USP15, a cytoplasmic DUB that accumulates at DSBs through interaction with BARD1 [37,92]. The constitutive turnover of BRCA1 is countered by USP9X, loss of which causes reduction in BRCA1 protein levels and reduced HR repair [93]. The BRCA1–BARD1 heterodimer constitutes an additional source of H2A ubiquitination in the DSB repair response, being responsible for mono-ubiquitination of the extreme C terminus of H2A at K125/127/129 [94]. This ubiquitination promotes chromatin alterations which facilitate long range DNA end resection [95]. This modification is regulated by USP48 which specifically cleaves mono-ubiquitinated H2A^K125/127/129Ub^. USP48 controls the extent of H2A^K125/127/129^ ubiquitination to prevent hyper-resection. In the absence of USP48, DNA is over-resected and the cells utilise the single strand annealing (SSA) sub-pathway in addition to HR. SSA is mutagenic due to the use of homologous repeats for bridging DSB ends, resulting in sections of DNA being deleted [48].

## CtIP

CtIP activity is essential for the early commitment to HR and is extensively regulated by both Ub and SUMO E3 ligases [96,97]. Ubiquitination and SUMOylation are important for regulating CtIP steady state, promoting recruitment to DSBs and limiting excessive resection. USP4 recruits to DSBs and interacts with both the MRN complex and CtIP [14,98]. In its inactive ubiquitinated form USP4 cannot interact with these factors but due to auto-de-ubiquitination these modifications are removed, enabling interaction with MRN-CtIP. It is not fully understood how USP4 regulates CtIP activity [14,98].

## EXO1

EXO1 is degraded after DSB induction as a means to limit hyper-resection [99]. EXO1 is SUMOylated by PIAS4 in response to DSB induction, which promotes its degradation independently of RNF4. SENP6 antagonises EXO1 SUMOylation promoting stabilisation [100]. UCHL5 is also required for BLM/EXO1 dependent end resection through de-ubiquitinating the INO80 subunit NFRKB, although the function of this subunit in regulating end resection is not currently understood [37].

## RPA complex

USP1 both directly and indirectly — through its partner UAF1 affects HR repair [101–103]. UAF1 is critical to the HR response through its association with RAD51AP1–RAD51–DNA complex which is required for efficient RAD51 loading and unloading [101–103]. The RAD51AP1 interaction is mediated through a SUMO-like domain, interestingly SUMO has previously been shown to be important for RAD51 foci formation [104]. The RPA70 subunit is SUMOylated in response to DSB induction due to the release of the constitutive interaction with SENP6. The SUMOylation of RPA70 improves interaction with RAD51 which is required for the exchange of RPA subunits for RAD51 during HR [105].

## BRCA2 and RAD51

Assembly of the BRCA1–PALB2–BRCA2 complex is restricted to S/G2 phases of the cell cycle. The Cullin3 adapter KEAP1 is responsible for preventing the formation of this complex outside of S/G2 by conjugating Ub on the interface between BRCA1 and PALB2. This disruptive ubiquitination is countered by USP11 [106,107]. USP11 turnover is regulated by KEAP1 in a cell cycle dependent manner, with USP11 protein levels at their lowest in G1. Thus cell cycle regulated turnover of USP11 allows a Ub dependent modification event to disrupt the formation of the BRCA1–PALB2–BRCA2 complex. This prevents RAD51 loading from occurring outside of S/G2 phases. Cells deficient of USP11 are defective in RAD51 loading due to failure in the formation of this complex, resulting in loss of HR [107,108]. USP21 interacts with, and stabilises the BRCA2–RAD51 complex by antagonising degradative BRCA2 ubiquitination. Down-regulation of USP21 results in failure of RAD51 loading and HR repair [109]. UCHL3 interacts with RAD51 and de-ubiquitinates residues that disrupt the RAD51–BRCA2 interaction. In the absence of UCHL3, RAD51 interaction with BRCA2 is disrupted, leading to reduced RAD51 foci formation and HR repair efficiency [110].

## Chromatin relaxation

USP8 forms a complex with the early DDR factor BRIT1. Under basal conditions BRIT1 is K63-Ub modified, which is antagonised by USP8. Low levels of BRIT1 K63-Ub modification are needed for recruitment to γH2AX which promotes the further recruitment of chromatin remodellers. The subsequent relaxation of chromatin then allows accumulation of downstream repair factors [111]. SENP7 maintains steady state SUMOylation levels of the chromatin component KAP1. ATM dependent phosphorylation of KAP1 is required to disrupt the SUMO dependent interaction with the CHD3 subunit of the NuRD complex. This disruption allows dispersion of the NuRD^CHD3^ complex from DSBs and subsequent chromatin relaxation [112]. In the absence of SENP7 the hyperSUMOylated KAP1 retains interaction with CHD3 resulting in failure of chromatin relaxation and HR repair [113]. The NuRD complex also recruits USP11 to DSBs promoting both histone de-ubiquitination and de-acetylation which enforces proper chromatin remodelling post DSB induction [65].

## Maintaining the free pool of Ub/Ubl for effective DSB response

Disruption of proteasome function results in attenuation of Ub driven DSB repair which can be overcome by suppling excess free Ub [62,114]. In addition to the proteasome, Ub/Ubl proteases are required for maintaining modifier availability, either by processing immature precursors or by recycling modifiers from substrates. Only six SUMO proteases are responsible for the bulk of SUMO maturation and recycling. Loss of SENP6 provokes a HR defect due to failure in RPA70 deSUMOylation and RAD51 filament formation [105], however, SENP6 is the major polySUMO2/3 protease, depletion of which causes substantial alterations in SUMO2/3 homeostasis in cells. These pleiotropic effects contribute to the HR defect, as re-supply of SUMO2 restores HR in SENP6 depleted cells [113]. Depletion of SENP2 also causes SUMO starvation resulting in HR failure that can be rescued with additional free SUMO [24].

## NEDDylation and DSB repair

NEDD8 conjugates are enriched at DSBs [115]. NEDD8 likely plays multiple roles in DSB resolution, but a role in H4 NEDDylation by RNF111, required for RNF168 accrual has been proposed [115]. To add further complexity RNF168 has also been proposed to NEDDylate H2A acting as a competitor for Ub signalling [116]. Additionally RNF111 NEDDylates CtIP and thus regulates DNA end-resection [117]. NEDDylation is reversed by just two known active proteases — the JAMM type DUB CSN5 within the COP9 signalosome and SENP8 [118]. Given the role of NEDDylation in the DSB repair response it is unsurprising that the COP9 signalosome and its DUB component CSN5 are recruited to DSBs in a NEDD8 dependent fashion. Disruption of this complex reduces HR [37,119].

## Ub/Ubl proteases and cancer

Imbalances in DSB associated Ub/Ubl protease activity can be caused by mutations or expression alterations. For example, inactivating mutations in BAP1 are common in mesothelioma, renal clear cell carcinomas and uveal melanomas. Cell lines carrying these mutations are particularly sensitive to ionising radiation and PARP inhibitors due to the important roles BAP1 plays in HR repair [68,69,120]. The amplification of the long arm of chromosome 3q is common in epithelial cancers, including lung squamous cell carcinoma, oesophageal, cervical and ovarian cancer. The 3q amplification encompasses the *RNF168*, *USP13* and *SENP2* genes. Over-expression of each of these individually promotes resistance to irradiation through altered Ub/SUMO signalling and DSB repair kinetics [24,89,121]. Several other DUBs are amplified in cancers including UCHL3 in breast cancer [110] and USP21 in hepatocellular carcinoma [109]. Dependency on Ub/Ubl proteases for cancer survival could make these useful targets in patient stratification. Indeed, inhibition of specific DUBs is currently being investigated as a means to enhance sensitivity to chemo/radiotherapies [122]. USP1 inhibitors have been proven effective in BRCA1 mutant tumours as USP1 is required for replication fork stability in the absence of functional BRCA1 [123]. The USP13/USP10 inhibitor Spautin-1 improves the anti-cancer activity of PARP inhibitors in a ovarian cancer model in mice [89]. USP7 inhibitors are also effective at sensitising therapy resistant CLL cells to HR directed therapies [124].

**Perspectives**

- The sheer number and lack of redundancy of DSB associated Ub/Ubl proteases highlights the complexity of Ub/Ubl signalling in genomic stability (summarised in Table 1). In many cases, the reduction or inactivation of a single Ub/Ubl protease is sufficient to entirely block DSB repair.
- The RNF8–RNF168–K63-Ub signalling node generates easily detectable DSB associated foci that can be visualised by a number of Ub specific antibodies such as FK2 and K63-Ub. As these modifications are read by 53BP1 which also forms readily detectable foci much of the initial research in the field focused on DUBs that regulate this step, indeed ∼8 DUBs have so far been identified that regulate 53BP1 dependent foci spreading [2]. However in more recent years Ub/Ubl proteases that regulate the earliest steps of DSB repair, the ordered clearance of repair factors and the later steps of RAD51 loading have been identified, suggesting Ub/Ubl modifiers are involved in multiple steps of DSB repair. Even greater nuance in Ub/Ubl modifier roles in DSB repair has been highlighted by a number of DUBs that remove disruptive Ub conjugates that impair protein–protein interactions needed for DSB repair.
- Further layers of complexity arise from the multiple Ub chain types now implicated in DSB repair. Additionally SUMOylation is unlikely to act separately from ubiquitination as co-modification and mixed chains are important signalling elements of the DSB response [125]. Therefore the diversity of chains types present at DSBs is likely many times greater than currently appreciated. SUMOylation is essential for the recruitment, activity and clearance of several DSB repair factors but we know relatively little concerning the activity of deSUMOylases in the DSB response, indeed there appears to be little redundancy between SENP enzymes as depletion of each causes specific DSB repair defects [24,105,113]. Finally, in both NHEJ and HR repair pathways there are multiple steps that are regulated by Ub/Ubls but the roles for their respective proteases await discovery.

**Table 1:** Summary table of the different roles played by Ub/Ubl proteases in the DSB response

Function	Ub/Ubl Protease
Ku dimer retention	UCHL3, OTUD5
MDC1 retention	SENP2, ATXN3, USP7
RNF8 stabilisation	ATXN3
RNF8–UBE2N catalysis antagonist	OTUB1
RNF168 stabilisation	USP34, USP7
RNF168 accumulation antagonist	A20, USP14
H2A/H2AXK13Ub spread antagonist	USP3, USP51, USP16
H2A-K118/119Ub antagonist	BAP1
K63-Ub/53BP1 spread antagonist	USP44, DUB3, USP11, ZUFSP, POH1, BRCC36, USP26, USP37
53BP1 spread (methyl dependent) antagonist	OTUB2
RAP80–BRCA1-A complex regulators	BRCC36, USP26, USP37, USP13, USP1-UAF1
BRCA1–BARD1 accumulation	USP15
BRCA1 stabilisation	USP9X
H2A-K125/K127/K129 antagonist	USP48
CtIP-MRN regulators	USP4
EXO1 stabilisation	SENP6, UCHL5
RPA–RAD51 interaction	USP1-UAF1, SENP6
BRCA2 stabilisation	USP21
RAD51 loading	USP11, UCHL3
Chromatin remodellers	USP8, SENP7, USP11
Free SUMO pool regulators	SENP2, SENP6

Note that many proteases play multiple roles in DSB signalling e.g. USP11.

## Abbreviations

DSBs: double strand breaks
HR: homologous recombination
NHEJ: non-homologous end joining
SSA: single strand annealing
